# A case of an eccrine spiradenoma in an elderly patient mimicking an epidermoid cyst

**DOI:** 10.1093/omcr/omaf103

**Published:** 2025-07-14

**Authors:** Meryeme Boutaarourt, Ouiame El Jouari, Salim Gallouj

**Affiliations:** Department of Dermatology-Venereology, Mohamed VI University Hospital, M3MF+GCG, 90100, La Nouvelle Ville Ibn Batouta, Tangier, Morocco; Faculty of Medicine and Pharmacy, M3MF+GCG, 90100, La Nouvelle Ville Ibn Batouta, Tangier, Morocco; Abdelmalek Essaadi University, 93030, BP2117 - Av. 9 Avril, Quartier M'hanneche II, Tetouan, Morocco; Department of Dermatology-Venereology, Mohamed VI University Hospital, M3MF+GCG, 90100, La Nouvelle Ville Ibn Batouta, Tangier, Morocco; Faculty of Medicine and Pharmacy, M3MF+GCG, 90100, La Nouvelle Ville Ibn Batouta, Tangier, Morocco; Abdelmalek Essaadi University, 93030, BP2117 - Av. 9 Avril, Quartier M'hanneche II, Tetouan, Morocco; Department of Dermatology-Venereology, Mohamed VI University Hospital, M3MF+GCG, 90100, La Nouvelle Ville Ibn Batouta, Tangier, Morocco; Faculty of Medicine and Pharmacy, M3MF+GCG, 90100, La Nouvelle Ville Ibn Batouta, Tangier, Morocco; Abdelmalek Essaadi University, 93030, BP2117 - Av. 9 Avril, Quartier M'hanneche II, Tetouan, Morocco

**Keywords:** eccrine spiradenoma, adnexal tumor, benign epithelial neoplasia, epidermoid cyst

## Abstract

Eccrine spiradenoma is one of the rarest benign adnexal tumors. Its clinical presentation is nonspecific, often necessitating a differential diagnosis with other benign cutaneous tumors, highlighting the crucial role of histological examination. The literature provides limited descriptions of this tumor, particularly in elderly patients, emphasizing the significance of our case. Our patient, a 61-year-old man with no significant medical history, presented with a painless cutaneous lesion on his back that had been progressively enlarging over six years. Clinical examination revealed a subcutaneous nodule with a firm consistency, measuring 2 cm in its largest dimension. Complete excision of the lesion was performed for pathological examination. Clinical findings, combined with histology and immunohistochemistry, confirmed the diagnosis of eccrine spiradenoma. This is a benign tumor of the eccrine sweat glands, rarely reported in the literature, typically presenting as a solitary nodule, sometimes multiple, with its primary clinical feature being paroxysmal tenderness. Early and complete surgical excision serves both diagnostic and therapeutic purposes. In addition to its rarity, the occurrence in an elderly patient and its painless nature make our publication particularly noteworthy. This article reports a case of eccrine spiradenoma identified in an elderly patient initially suspected of having an epidermoid cyst, underscoring the importance of histopathological examination for any cutaneous tumor.

## Introduction

The eccrine spiradenoma is a rare benign adnexal tumor that develops from the epithelium of cutaneous eccrine glands. It was first described by Kersting and Helwig in 1956. This tumor can occur at any age, but it predominantly affects young adults with no sex predilection.

It typically presents as a solitary, rounded, firm, and sometimes painful dermal nodule with slow growth that gradually progresses over time. It is most commonly located on the cephalic region of the body, particularly on the trunk, but it can also be found on the upper and lower extremities, especially on the dorsal surface.

The diagnosis is initially suggested clinically; however, due to its nonspecific clinical features, confirmation requires excision followed by histological analysis.

Given its rarity, we report a case of eccrine spiradenoma in an elderly patient that posed a differential diagnostic challenge with an epidermoid cyst.

## Case report

We report the case of a 61-year-old patient with no significant medical history, presenting for the past six years with a painless, non-pruritic cutaneous lesion on the back that has progressively increased in size over time. The lesion developed in an afebrile context with preserved general health.

**Figure 1:**
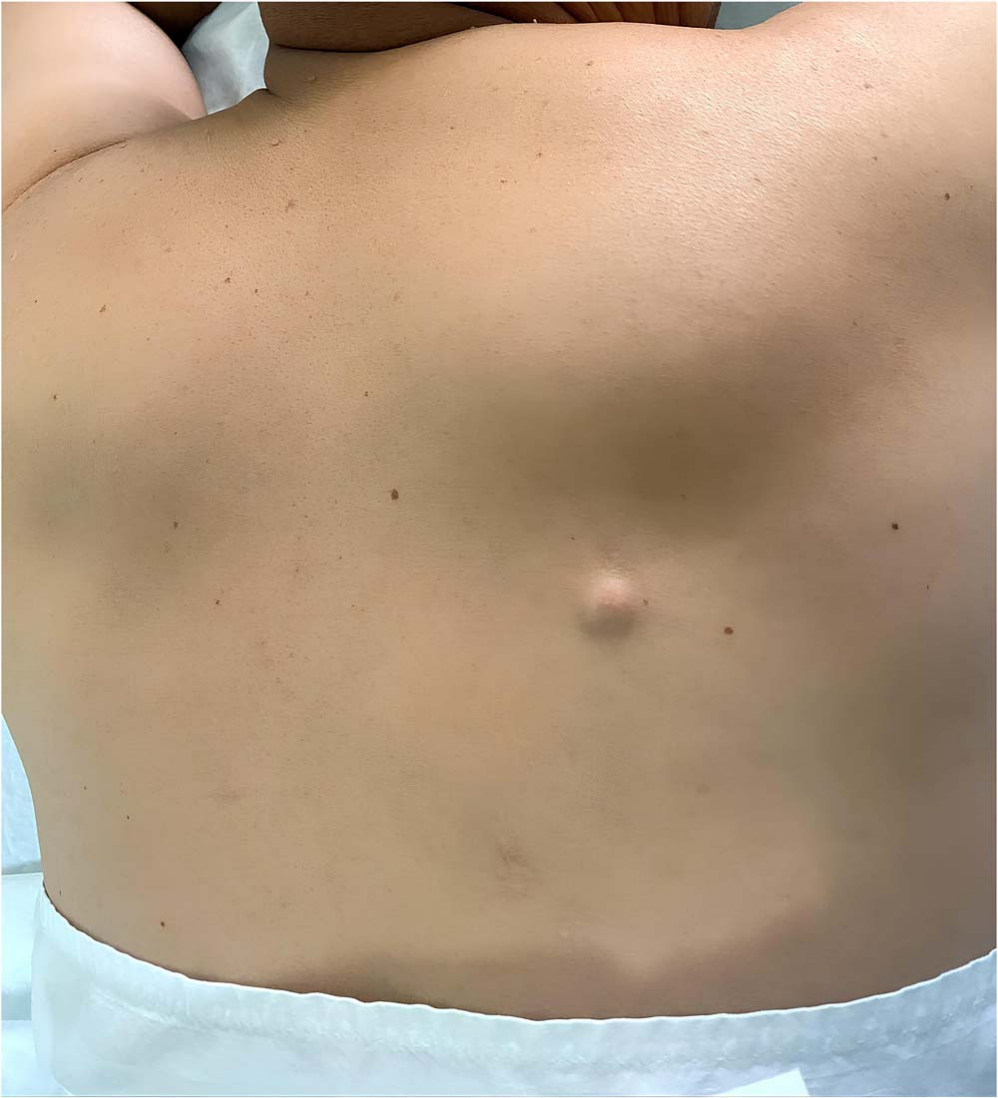
Clinical appearance of the lesion showing a subcutaneous nodule on the back, measuring 3 cm in long axis.

Clinical examination revealed a subcutaneous nodule, firm in consistency, mobile in relation to the deeper plane but fixed to the superficial plane, measuring 3 cm in its largest diameter (Fig. 1). The initial clinical impression suggested an epidermoid cyst.

**Figure 2:**
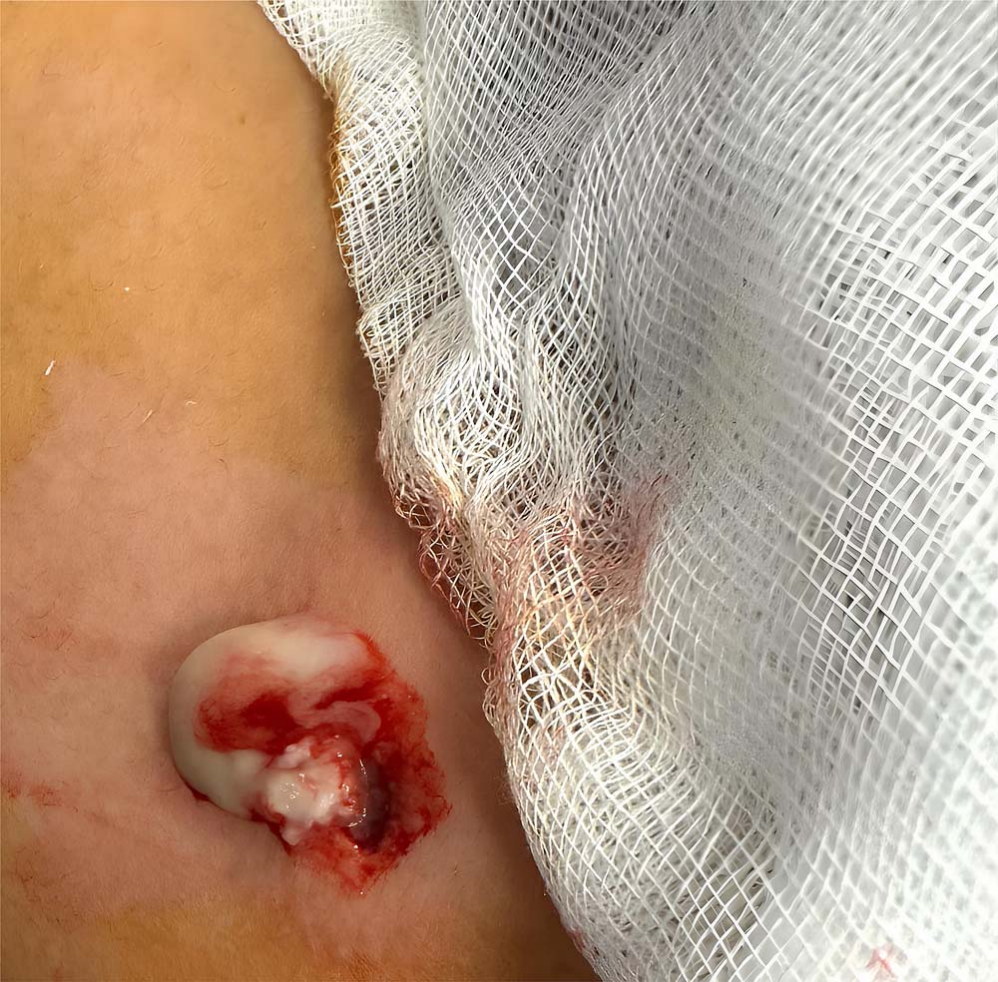
Complete excision of the lesion including the peripheral capsule.

A complete excision of the lesion, including the peripheral capsule, was performed for histopathological examination (Fig. 2).

**Figure 3:**
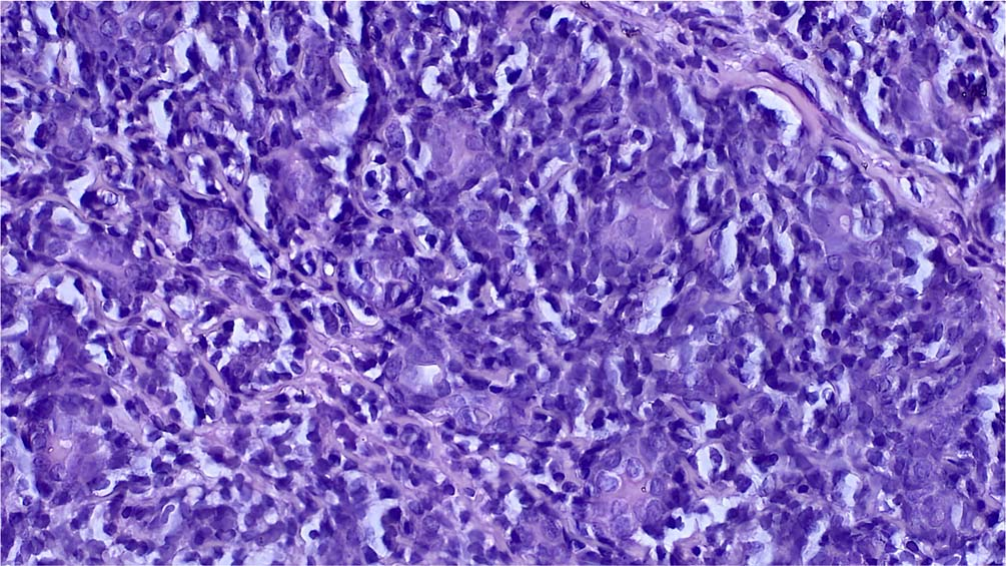
Histological section at high magnification (×40) showing a population of small basaloid cells with hyperchromatic nuclei, associated with larger cells with vascular nuclei. The stroma is scanty and shows slight inflammation.

**Figure 4:**
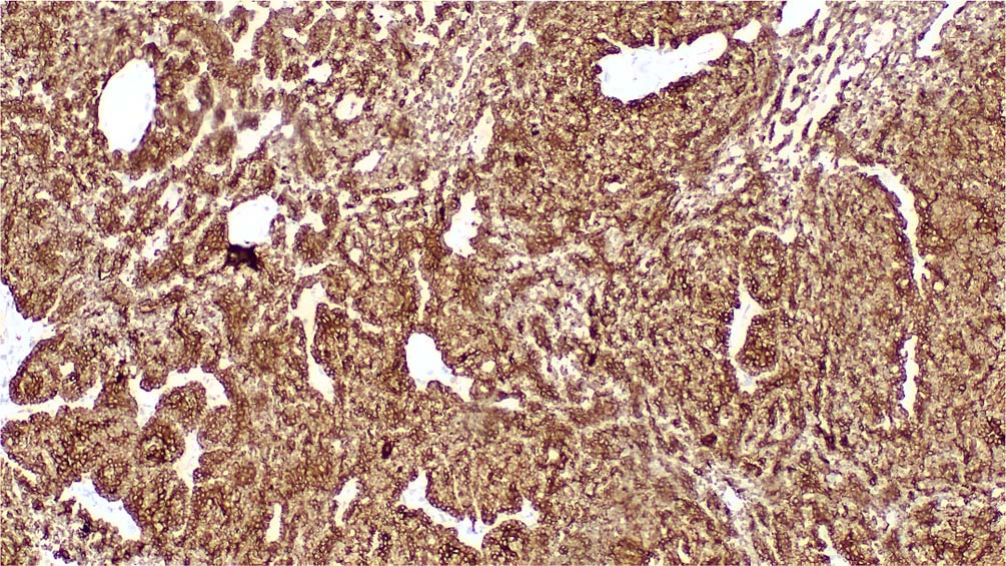
Strong and diffuse cytoplasmic marking of tumor cells by the anti-cytokeratin AE1/AE3 antibody.

Histological analysis revealed a benign tumor proliferation unconnected to the epidermis, arranged in diffuse sheets and small glandular structures. It was composed of small basaloid cells exhibiting mild cytonuclear atypia, with the presence of eosinophilic hyaline deposits in the form of droplets and bands, along with a mild scattered lymphocytic inflammatory infiltrate (Fig. 3). The stroma was thin and harbored diffuse vascular proliferation with swollen endothelial cells. Immunohistochemical analysis showed a strong, diffuse cytoplasmic staining with AE1/AE3 antibodies (Fig. 4).

The clinical findings, coupled with histology and immunohistochemistry, confirmed the diagnosis of eccrine spiradenoma.

## Discussion

### Pathophysiology

Although the exact etiology of eccrine spiradenomas remains unknown, they appear to be caused by a defective tumor suppressor gene. In Brooke-Spiegler syndrome (BSS), which is characterized by multiple adnexal tumors, including cylindromas, spiradenomas, and trichoepitheliomas, a defect in the CYLD gene located on chromosome 16 at position 12.1 is typically observed [1].

Currently, it is understood that the pathogenesis is linked to the differentiation of the secretory segment of eccrine sweat glands [2], and the pain associated with these tumors is attributed to small, unmyelinated axons penetrating the hyaline stromal sheath.

### Cellular origin and differentiation

Eccrine spiradenoma is a tumor derived from epithelial cells of sweat glands. These cells differentiate into two main populations: *basaloid cells* which are small with hyperchromatic nuclei and scant cytoplasm, forming the nodular structure. And *secretory cells* which are larger with clear nuclei and abundant cytoplasm, resembling the secretory cells of eccrine or apocrine glands.

Research suggests that spiradenomas may originate not only from eccrine glands but also from folliculosebaceous units or apocrine glands, given the presence of apocrine markers such as cytokeratin 7.

### Molecular and genetic mechanisms

A mutation in the CYLD gene, located on chromosome 16q12.1, plays a pivotal role. This gene is a tumor suppressor involved in the regulation of the NF-κB signaling pathway. Its mutation leads to excessive activation of this pathway, promoting cellular proliferation and inhibiting apoptosis. This mechanism is particularly implicated in syndromes such as Brooke-Spiegler syndrome, where patients develop multiple spiradenomas, cylindromas, and trichoepitheliomas [3].

### Tumor microenvironment

The interaction between tumor cells and inflammatory cells (lymphocytes and plasma cells) is a notable feature. Intratumoral lymphocytes within the tumor play an immunomodulatory role and may influence tumor growth. This inflammatory infiltration is partially attributed to the expression of pro-inflammatory cytokines induced by the abnormal activation of NF-κB [4].

### Structural pathophysiology

Eccrine spiradenoma is a well-encapsulated tumor located within the dermis. Its nodular architecture reflects the cellular heterogeneity of normal sweat glands, characterized by: disorganized proliferation of ductal and secretory cells, pseudo-glandular or duct-like structures surrounded by fibrous stroma and central necrosis in larger lesions, resulting from rapid tumor growth exceeding its vascular supply.

### Progression and malignant transformation

While benign, eccrine spiradenoma can, in rare cases, evolve into spiradenocarcinoma. Malignant transformation is associated with progressive accumulation of additional mutations and increasing cytogenetic abnormalities that favor local invasion and potential metastatic dissemination.

## Clinical diagnosis and implications for elderly patients

Eccrine spiradenoma is a rare benign tumor arising from sweat glands, specifically from eccrine differentiation. It affects both sexes equally and can occur at any age but is more common in young adults under the age of 40. However, its occurrence in elderly patients, as illustrated in the present case, represents a notable exception, posing unique diagnostic and therapeutic challenges due to atypical presentations, an increased risk of complications, and the frequent presence of comorbidities in this population.

Clinically, the lesion most often presents as a solitary tumor (in 97% of cases) larger than 1 cm in diameter (potentially reaching up to 5 cm) [5], well-defined, flesh-colored or pink to bluish, firm in consistency, and often painful with paroxysmal episodes. This can lead to diagnostic confusion with musculoskeletal conditions or other dermatological conditions such as a compressive lipoma, an infected epidermoid cyst, or an inflammatory cylindroma, thereby delaying diagnosis. However, the characteristic pain was absent in our patient, adding further complexity to the diagnostic process. The tumor can appear on any part of the body, with a preference for the trunk, though 20% of cases are reported on the extremities [6].

Despite its predominantly benign nature, eccrine spiradenoma can undergo malignant transformation into spiradenocarcinoma, particularly in patients over 50 years of age [7]. Approximately 50 cases of this transformation have been documented in the literature [1], highlighting significant aggressiveness, a considerable risk of metastasis (around 50%) [6] to lymph nodes, bones, lungs, and the brain, and a mortality rate ranging from 20% to 39%.

Warning signs suggesting malignancy include the onset of previously absent pain, increased tenderness, changes in color, rapid growth, bleeding, or ulceration of a lesion that had been stable for a long period [5], emphasizing the importance of early diagnosis.

It is worth noting that elderly patients are more likely to exhibit complex clinical presentations, including multiple nodules or abnormally rapid growth, which may indicate malignant transformation.

## Histopathological and immunohistochemical diagnosis

### Histopathology

Histologically, eccrine spiradenoma presents as one or more well-demarcated basophilic intradermal lobules located within the dermis and subcutaneous tissue. These lobules are surrounded by an abundant eosinophilic fibrous capsule without connection to the epidermis.

Under magnification, two distinct types of basaloid cells are visible [5]: *basaloid cells* which are small, dark cells with compact hyperchromatic nuclei, located at the periphery of the lobules. And *clear cells* which are larger with pale cytoplasm and oval acidophilic nuclei, primarily located at the center of the lobules.

Tubular differentiation may be present, with canal-like structures lined by acidophilic epithelial cells. A lymphocytic infiltrate and prominent telangiectasia are also frequently observed. Notably, mitoses are absent.

### Immunohistochemistry

Immunohistochemistry plays a crucial role in differentiating eccrine spiradenoma from other subcutaneous tumors, such as epidermoid cysts, cylindromas, and adnexal carcinomas [8]. It demonstrates: AE1/AE3; positive, confirming epithelial origin; these antibodies, markers of pan-cytokeratins, show diffuse positivity in tumor cells, affirming their epithelial origin. CK5/6 and CK7/8; positive, indicating glandular differentiation. EMA (Epithelial Membrane Antigen); positive staining of glandular and ductal structures. S100 protein; occasionally weakly positive in certain zones, particularly in peripheral myoepithelial cells. P63; focally positive in basal cells, indicating a myoepithelial origin. SMA (Smooth Muscle Actin); often negative, aiding in distinguishing eccrine spiradenoma from other tumors with myoepithelial cells. CEA (Carcinoembryonic Antigen); negative or weakly expressed. CD10; occasionally expressed but nonspecific.

The expression of cytokeratins such as AE1/AE3 is a key criterion for confirming epithelial origin in the differential diagnosis with other dermal or adnexal tumors. However, the definitive diagnosis requires consideration of histological features, such as glandular architecture with peripheral basophilic cells and larger clear cells at the center. This differentiates eccrine spiradenoma from epidermoid cysts, which exhibit a stratified keratinized layer and a central keratin-filled lumen. Immunohistochemical markers such as S100, EMA, and CK7, absent in epidermoid cysts, further support the diagnosis.

## Differential diagnosis

Eccrine spiradenoma shares clinical and histological features with several other benign and malignant cutaneous tumors, including:

### Epidermoid cyst

The epidermoid cyst and eccrine spiradenoma share some clinical similarities but differ in origin, histology, and clinical presentation. The common features are mainly the *nodular appearance*; both present as well-demarcated subcutaneous nodules, the *location*; epidermoid cysts can appear on the face, trunk, or limbs, which are also common sites for eccrine spiradenomas, and the palpation; both lesions can be firm, mobile, and rounded on palpation. Regarding clinical and histological differences; *clinically*, spontaneous pain is a hallmark of eccrine spiradenoma, while epidermoid cysts are usually asymptomatic. Keratinous content may occasionally drain from an infected cyst, a feature absent in spiradenomas. Imaging (ultrasound or MRI) is also useful; ultrasound helps differentiate a solid mass (spiradenoma) from a cystic lesion, while *MRI* can show enhancement of the spiradenoma after gadolinium injection, which is rare for an epidermoid cyst. As for *histology*, the glandular architecture is characteristic of spiradenoma.

Although their similar nodular appearance may cause confusion, the adnexal origin and distinct histological and immunohistochemical characteristics of eccrine spiradenomas differentiate them from epidermoid cysts. Moreover, the absence of a fistulous opening, a usual feature of an epidermoid cyst, should prompt consideration of other diagnoses.

**Table:** 

Criteria	Eccrine spiradenoma	Epidermoid cyst
Clinical presentation	- Firm, well-defined nodule, often painful on palpation.—Typically located on the trunk, head and neck.—Slow-growing and usually solitary.—Smooth surface, sometimes with a bluish or erythematous tint.	- Firm, subcutaneous nodule, painless (except in case of infection).—Most commonly found on the face, scalp, back, or genital regions.—Contains a cavity filled with keratin, sometimes with a central pore.
Histology	- Proliferation of basaloid cells arranged in well-defined lobulated nests.—Two cell types: peripheral dark cells and central clear cells.—Presence of tubular structures with eccrine differentiation.—Hyalinized and highly vascularized stroma with lymphocytic infiltration.	- Cavity lined with stratified squamous epithelium without atypia.—Absence of glandular structures or eccrine differentiation.—Lamellar keratinized content without pilo-sebaceous appendages.
Imaging	- Ultrasound: Hypoechoic mass with well-defined margins, sometimes with posterior acoustic enhancement.—Doppler: Possible hypervascularization (due to inflammatory stroma).—MRI: Intermediate signal on T1, hyperintense on T2 with enhancement after gadolinium injection (stromal vascularization).	- Ultrasound: Hypoechoic mass with posterior acoustic enhancement.—Doppler: No significant vascular signal.—MRI: Variable signal depending on keratinized content, generally iso- or hyperintense on T2, with little to no enhancement.

### Cylindroma

This is a benign tumor of sweat glands often confused with eccrine spiradenoma due to histological similarities. Some studies suggest that spiradenomas and cylindromas may represent two ends of a spectrum of dermal tumors derived from a common progenitor. Common features include the *origin*; both arise from sweat gland adnexal structures (eccrine or apocrine glands), the *clinical presentation* which consists of well-defined and sometimes painful dermal nodules or masses, with the head and neck as the predominant *location*. The *evolution* which is generally low and benign, but with a rare potential for malignant transformation. Regarding clinical and histological differences; cylindromas presents clinically, most often, as multiple nodules, often on the scalp, and are rarely painful unless large. Histologically, this tumor consists only of basaloid cells arranged in clusters, surrounded by a homogeneous hyalinized stroma, unlike spiradenomas, which also feature clear cells. *In imaging*, spiradenoma may show enhancement after gadolinium injection on MRI due to its vascularized stroma, whereas cylindroma generally has less vascularization and little to no enhancement.

**Table:** 

Criteria	Eccrine spiradenoma	Cylindroma
Clinical presentation	- Firm, well-defined nodule, often painful on palpation.—Commonly located on the head, neck and trunk.—Slow-growing and usually a single lesion.—Smooth surface, sometimes with a bluish or erythematous appearance.	- Usually multiple dome-shapes nodules (especially in familial cases).—Most frequently affects the scalp and forehead, forming a turban tumor when extensive.—Firm, well-circums cribed masses, painless unless inflamed.—Smooth or lobulated surface, pink or flesh-colored.
Histology	- Well-defined lobular proliferation of basaloid cells arranged in nests.—Two cell types: dark peripheral cells and pale central cells.—Presence of eccrine ductal structures.—Hyalinized, vascularized stroma with lymphocytic infiltration.	- Jigsaw puzzle-like architecture with rounded islands of basaloid cells surrounded by a thick hyalinized stroma.—No clear separation between dark and pale cells.—Often coexists with spiradenoma (spiradenocylindroma).—Lack of significant inflammatory infiltrate.
Imaging	- Ultrasound: Hypoechoic solid mass, well-defined, sometimes with posterior acoustic enhancement.—Doppler: Possible vascular signal due to inflammatory stroma.—MRI: Intermediate signal on T1, hyperintense on T2 with enhancement after gadolinium injection.	- Ultrasound: Well-circums cribed hypoechoic nodule, sometimes with internal septations.—Doppler: Poor vascularization compared to spiradenoma.—MRI: Intermediate to low signal on T1, variable T2 intensity, minimal or no enhancement after contrast.

### Trichoepithelioma

A benign tumor derived from hair follicles, it may share similar histological features, requiring detailed analysis for differentiation. *Clinically*; trichoepitheliomas present as multiple painless nodules, often on the face or sebaceous areas. *Histologically*, basaloid cells are arranged in nests with palisading borders, accompanied by fibroblastic, sometimes myxoid, stroma and keratinized horn cysts. On *immunohistochemistry*; CK7, CEA, EMA, and AE1/AE3 are negative, whereas BER-EP4 is positive.

**Table:** 

Criteria	Eccrine spiradenoma	Trichoepithelioma
Clinical features	- Solitary, firm, well-defined nodule.—Often painful on palpation.—Commonly located on the head, neck and trunk.—Slow-growing and usually isolated.—Smooth surface, sometimes with a bluish or erythematous appearance.	- Typically multiple, small firm well-defined nodules.—Painless, slow growing.—Mainly located on the face (eyebrows, cheeks, nose).—Smooth or slightly verrucous surface, flesh-colored or translucent.
Histology	- Lobular proliferation of basaloid cells in well-defined nests.—Two cell types: dark peripheral cells and pale central cells.—Presence of eccrine ductal structures.—Hyalinized, vascularized stroma with lymphocytic infiltration.	- Proliferation of small basaloid nests in a fibrous stroma.—Presence of follicular differentiation, resembling embryonic hair follicles.—Presence of horn cysts and keratinization.—No eccrine glandular structures.
Imaging	- Ultrasound: Hypoechoic solid mass, well-defined, sometimes with posterior acoustic enhancement.—Doppler: Possible vascular signal due to inflammatory stroma.—MRI: Intermediate signal on T1, hyperintense on T2 with enhancement after gadolinium injection.	- Ultrasound: Hypoechoic, multiple small nodules, well-defined.—Doppler: Little to no vascularization.—MRI: Intermediate to low signal on T1 and T2, with minimal or no enhancement after gadolinium injection.

### Spiradenocarcinoma (malignant transformation of spiradenoma)

Clinically, it presents as a painful, rapidly growing, sometimes ulcerated or infiltrative nodule, often on a pre-existing spiradenoma. Histology shows cellular atypia, frequent mitoses, and invasion of surrounding tissues.

**Table:** 

Criteria	Eccrine spiradenoma	Spiradenocarcinoma
Clinical features	- Solitary, firm, well-defined nodule.—Often painful on palpation.—Commonly located on the head, neck and trunk.—Slow-growing and usually isolated.—Smooth surface, sometimes with a bluish or erythematous appearance.	- Malignant transformation of a pre-existing spiradenoma (in 20–30% of cases).—Rapidly growing, poorly defined mass.—Can be painful with ulceration, bleeding, or inflammation.—Preferentially found on the head, neck, trunk.—Possible extension to surrounding tissues and lymphadenopathy.
Histology	- Lobular proliferation of basaloid cells in well-defined nests.—Two cell types: dark peripheral cells and pale central cells.—Presence of eccrine ductal structures.—Hyalinized, vascularized stroma with lymphocytic infiltration.	- Loss of lobular architecture with nuclear atypia, marked pleomorphism, and frequent mitoses.—Common tumor necrosis.—Progressive loss of eccrine ductal structures.—Infiltration of surrounding tissues and possible vascular emboli.
Imaging	- Ultrasound: Hypoechoic solid mass, well-defined, sometimes with posterior acoustic enhancement.—Doppler: Possible vascular signal due to inflammatory stroma.—MRI: Intermediate signal on T1, hyperintense on T2 with enhancement after gadolinium injection.	-Ultrasound: Heterogeneous, poorly defined mass, possible necrotic areas.—Doppler: Increased, disorganized vascularization.—MRI: Heterogeneous enhancement after gadolinium injection, necrotic zones, and surrounding tissue infiltration.—CT scan: Used to assess locoregional extension and detect pulmonary or lymph node metastases.

Eccrine spiradenomas may also be mistaken for other subcutaneous tumors, such as glomus tumors or angioleiomyomas, due to their pain and prominent vascularization [8]. Immunohistochemical studies can guide the diagnosis.

Differentiating eccrine spiradenomas from other adnexal or cutaneous tumors can be particularly challenging in elderly patients due to: *aging skin*; thinner and atrophic skin complicates clinical differentiation of eccrine lesions, *increased malignant potential*; benign pre-existing tumors in older patients are more prone to malignant transformation, *comorbidities*; conditions such as immunosuppression and chronic sun exposure can alter the clinical and histological presentation. In addition to an *atypical localization and presentation*; spiradenomas in elderly patients may appear in unusual locations such as the breast or nipple, often presenting as solitary, sometimes painful nodules with a bluish, gray, or violet hue. Considering these factors during diagnostic evaluation is essential. However, histopathological and immunohistochemical analysis remains the gold standard for accurate diagnosis.

## Treatment and management

Surgical excision remains the gold standard for treatment, with low recurrence rates documented when resection is complete [6]. However, incomplete surgical removal carries a high risk of local recurrence.

Management of spiradenomas in elderly patients presents specific challenges, requiring consideration of several factors: first, *associated comorbidities*; chronic conditions such as diabetes, hypertension, or cardiovascular disorders can complicate surgical interventions. *Healing and recovery*; age-related skin changes, such as decreased elasticity and thickness, can affect aesthetic outcomes following surgical excision. Rigorous postoperative care is therefore essential to prevent complications. *Risk of recurrence*; incomplete excision may lead to local recurrence, necessitating additional interventions. These factors call for meticulous planning and thorough discussions with patients regarding postoperative expectations. In cases of malignant transformation, a more aggressive therapeutic approach is recommended, including wide excision and, if necessary, adjuvant treatments, especially in the presence of metastases. In such cases, lymph node dissection may be considered, radiotherapy and chemotherapy have shown limited effectiveness, although some cases have reported a favorable response to hormone therapy, particularly when hormone receptors are positive [9].

In summary; in elderly patients, careful assessment of comorbidities and healing capacity is vital to ensure optimal outcomes. Additionally, the psychosocial impact of eccrine spiradenomas in this demographic, particularly due to chronic pain, aesthetic concerns, and fears of malignant transformation. These aspects must be carefully considered as part of the patient’s comprehensive management plan.

## Prognosis and follow-up

The prognosis of eccrine spiradenoma is generally good. It is a benign tumor with no metastatic potential, and its evolution is most often stable over several years. Growth is slow, and pain observed in some cases is related to the rich innervation of the tumor. Nevertheless, the prognosis may be more guarded in elderly patients due to the risk of malignant transformation into spiradenocarcinoma, especially after a long duration of progression. In such cases, the prognosis depends on several factors [10]; particularly the patient's age, which averages 59 years, with reported cases up to 92 years old; the size of the lesion, which averages 3.9 cm at the time of diagnosis; and the location of the tumor, most commonly on the trunk and extremities, as well as the presence of metastases. These are associated with a significant recurrence rate (around 17.5%) after surgical excision and may affect regional lymph nodes, lungs, brain, and liver. Distant metastases, although rare, are associated with a poor prognosis and a significant mortality rate [10].

Increased vigilance is therefore essential to monitor for warning signs that could indicate progression to spiradenocarcinoma; first, rapid clinical changes such as a sudden increase in lesion size, which contrasts with the usual slow growth of benign spiradenomas, and the appearance of ulcerated areas or spontaneous bleeding. Exacerbated pain: while benign spiradenomas are often painful on palpation, persistent and increasing pain could suggest malignant transformation. Unusual morphological changes, such as the appearance of irregular or infiltrative borders and progressive induration around the tumor. Fixation and adherence to deep structures: reduced mobility of the tumor relative to underlying tissues may be a sign of local invasion. Furthermore, the presence of disproportionate inflammatory signs such as marked erythema, edema, or localized heat may suggest aggressive tumor proliferation [11].

Fortunately for our patient, none of these alarming signs were present. However, in the case of a diagnosed eccrine spiradenoma in an elderly patient, surveillance must be rigorous. It is all the more important as certain forms may be overlooked or mistaken for other benign skin lesions. It is based primarily on regular clinical examination (every 6 to 12 months), which is crucial for detecting any recurrence or suspicious change (rapid increase in size, pain, ulceration); additional imaging (ultrasound, MRI) should be performed in case of doubt about local extension or tumor recurrence. A wide re-excision is indicated if histological criteria suggest malignant transformation (presence of cellular atypia, numerous mitoses, infiltration of adjacent tissues).

Although eccrine spiradenoma is generally benign, its malignant transformation into spiradenocarcinoma, particularly in elderly individuals, requires rigorous management and careful monitoring to optimize prognosis.

## Conclusion

Eccrine spiradenomas, though rare, should be considered in the differential diagnosis of cutaneous tumors, particularly in atypical demographics such as the elderly.

Paroxysmal pain is highly suggestive, but painless presentations, as seen in our patient, are also possible.

Early diagnosis is critical due to the potential progression to a rare but potentially fatal malignant tumor, most commonly observed in the multiple variant.

Detailed histopathological and immunohistochemical analysis is essential for effective management and to prevent complications associated with malignant forms.

Early and complete surgical excision serves both as a diagnostic tool and a curative therapeutic option.

In elderly individuals, eccrine spiradenomas require particular attention due to their variable clinical presentation and risk of malignant transformation. A multidisciplinary approach, including close clinical monitoring, tailored surgical excision, and management of comorbidities, is crucial to ensure a favorable prognosis.
